# Supplementary material for: Glycolysis regulates Hedgehog signalling via the plasma membrane potential
Source: EMBO J. 2020 Oct 6;39(21):e101767. doi: 10.15252/embj.2019101767 (PMC7604625; doi:10.15252/embj.2019101767)
Supplement: Supplementary file 1 — Appendix [file EMBJ-39-e101767-s001.pdf]

APPENDIX

Table of contents

Appendix Fig S1-S6…………………………………………………………………2-7
Appendix Figure legends…………………………………………………………..8-12
Appendix Tab S1-S3……………………………………………………………...…13-20

Appendix Fig S1

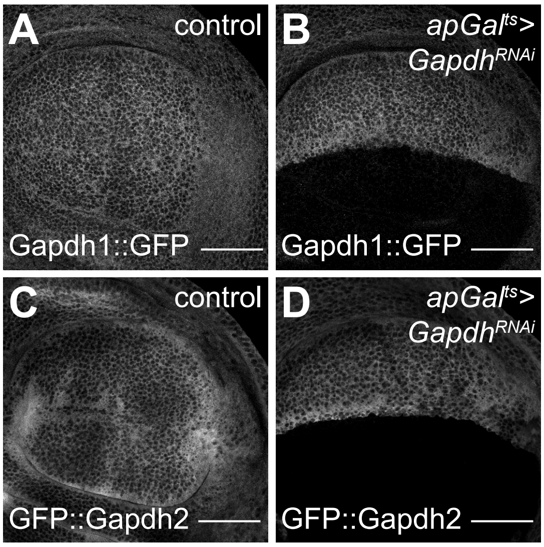

Appendix Fig S2

A Significant change of wing blade area, but not aspect ratio

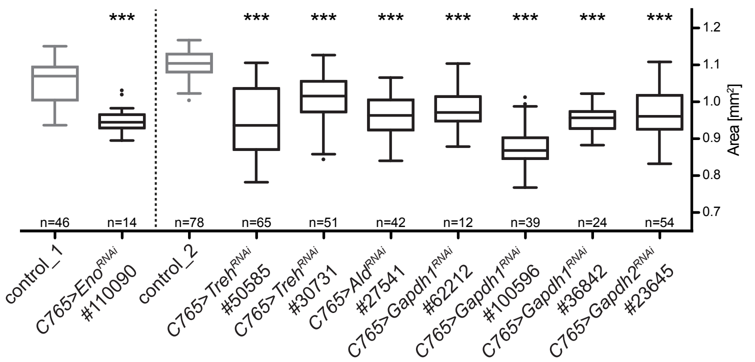

B Significant decrease in wing blade aspect ratio

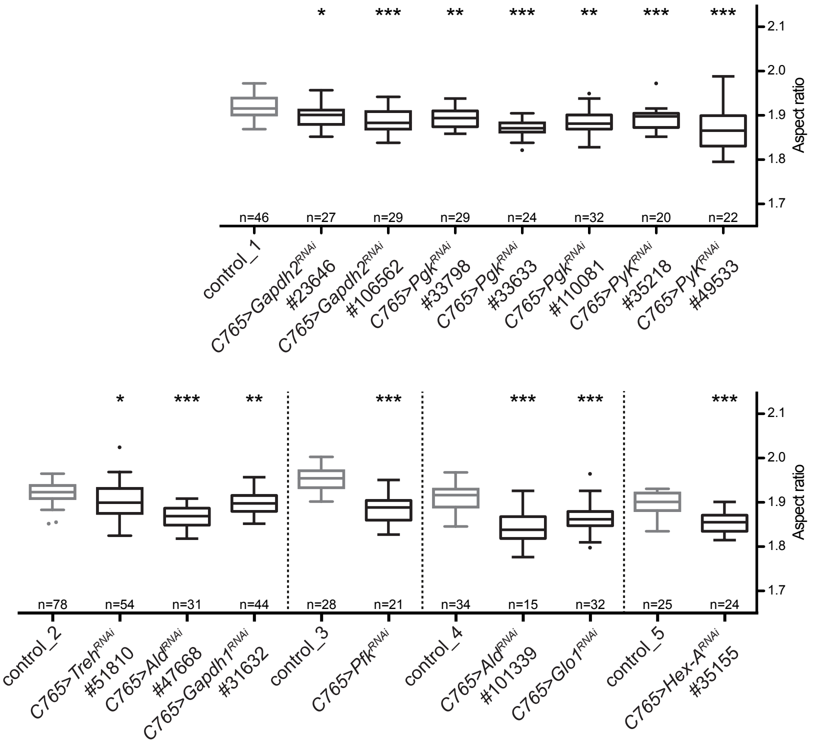

Appendix Fig S3

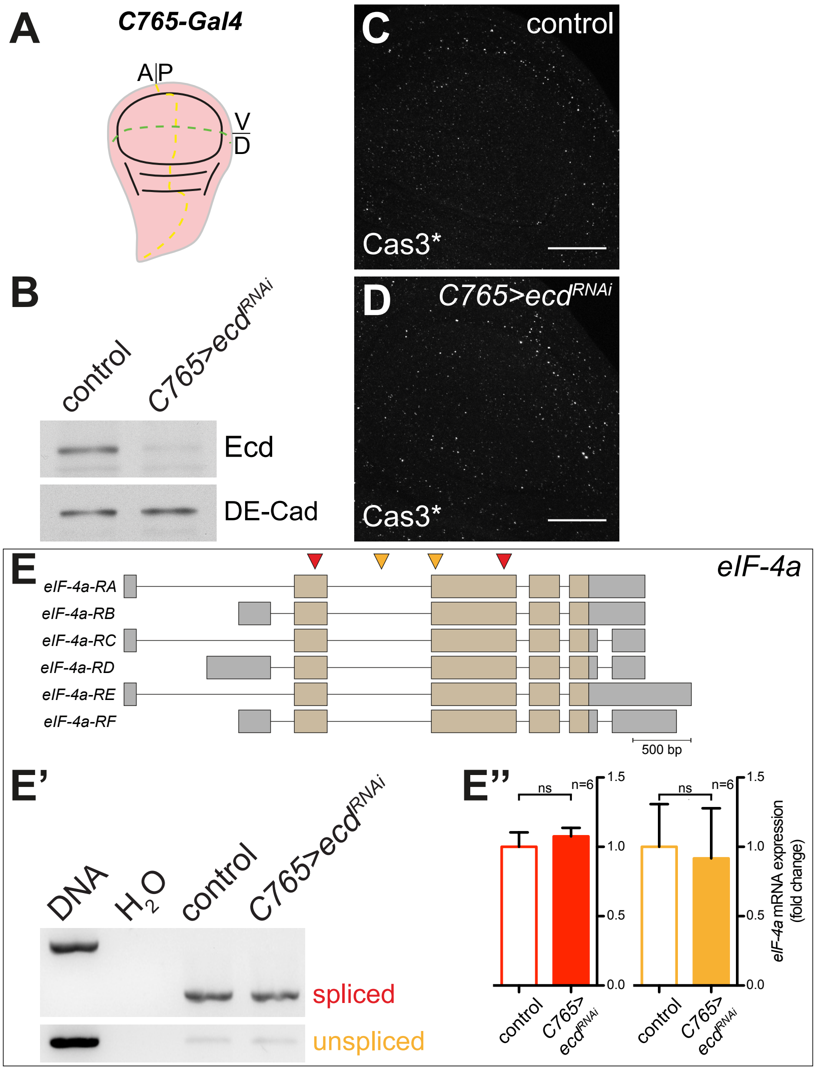

Appendix Fig S4

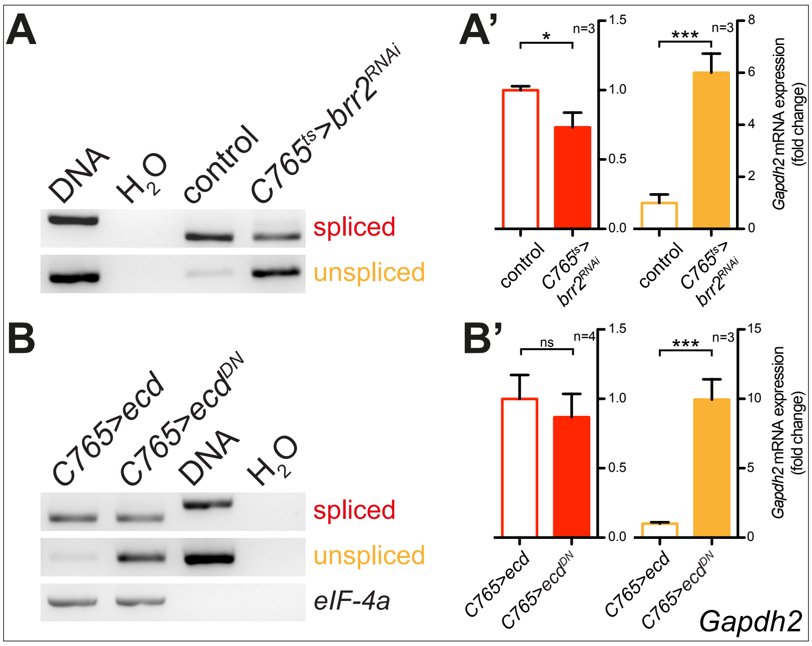

Appendix Fig S5

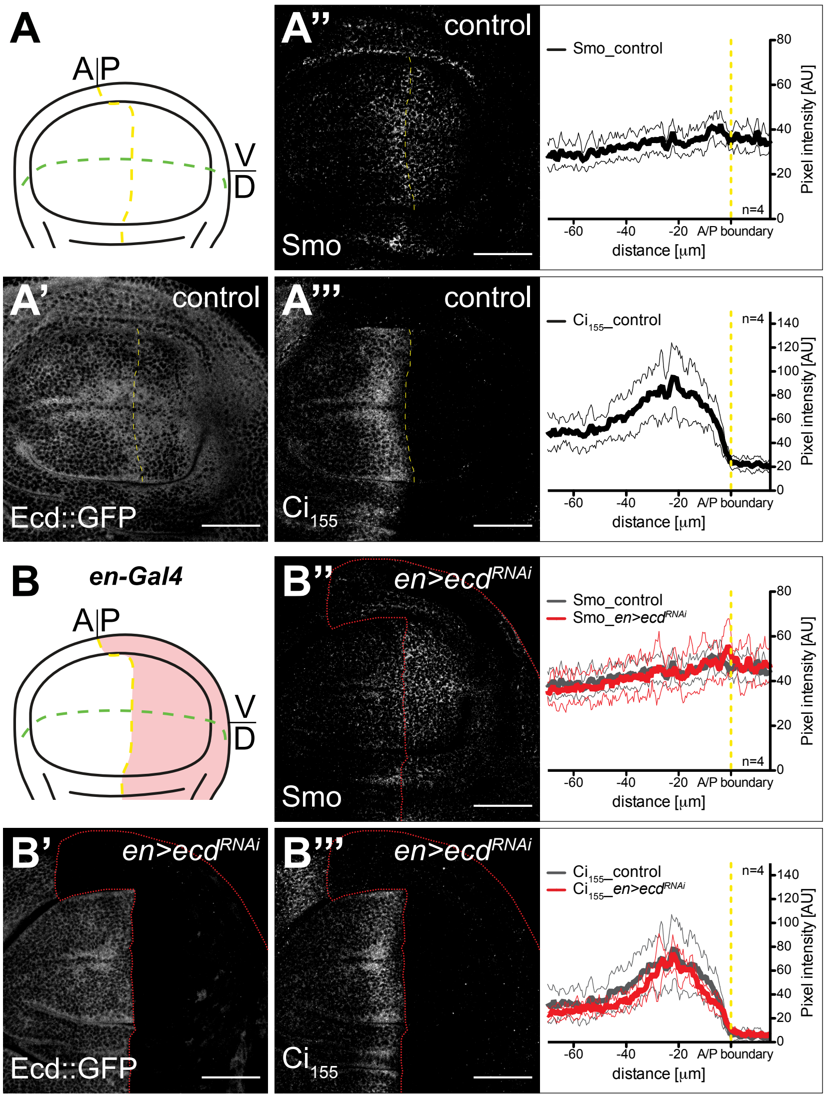

Appendix Fig S6

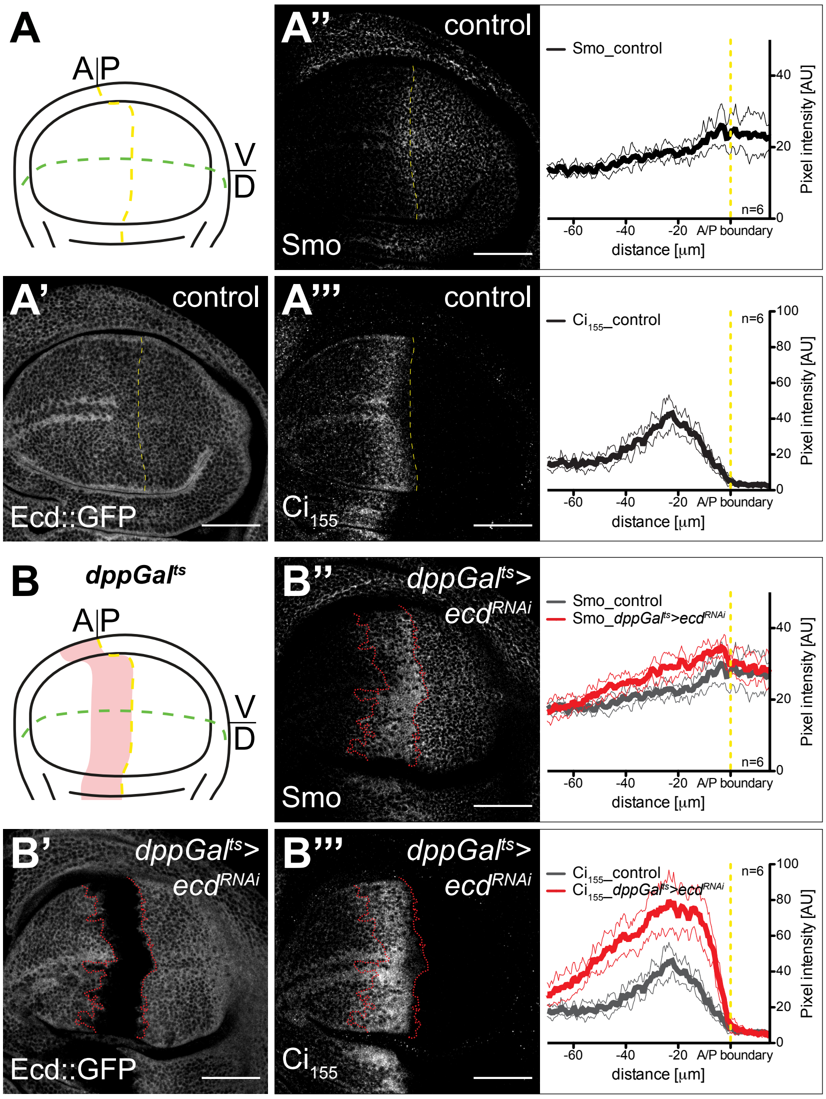

Appendix Fig S1. *Gapdh*^{RNAi} depletes both Gapdh1 and Gapdh2

A-D Time-controlled knock-down of *Gapdh1* and *Gapdh2* in the dorsal compartment of the wing disc using *apGal*^{ts}>*Gapdh*^{RNAi} (see Fig 1C for the expression pattern of *apGal*^{ts}). IF of control and *apGal*^{ts}>*Gapdh*^{RNAi} wing discs, stained for either Gapdh1::GFP (A, B) or GFP::Gapdh2 (C, D) using an anti-GFP antibody. Wing discs were analyzed 120 h after RNAi induction. Scale bars= 50 μm.

Appendix Fig S2. Knock-down of metabolic enzymes affects adult wing blade area and shape

A Tukey box-and-whiskers plot showing quantification of the area of control wings and wings in which RNAi against different metabolic enzyme was induced throughout wing development using *C765-Gal4* (see Appendix Fig S3A for the expression pattern of *C765-Gal4*). RNAi induction using these different Bloomington and VDRC lines (see Materials and Methods) resulted in a significant decrease in wing blade area but not in wing blade aspect ratio. Lower and upper hinges correspond to the first and third quartiles, vertical lines extend to ± 1.5 times the interquartile range. Sample size is indicated for each RNAi line. Knock-down experiments were conducted in groups, each including its own control (control_1 and control_2). Statistical analysis was performed using one-way ANOVA, followed by Dunnett's multiple comparisons test. ***$p \leq 0.001$.

B Tukey box-and-whiskers plot showing quantification of the shape (aspect ratio, major:minor axis) of control wings and wings in which RNAi against different metabolic enzyme was induced throughout wing development using *C765-Gal4* (see Appendix Fig S3A for the expression pattern of *C765-Gal4*). RNAi induction using these different Bloomington and VDRC lines (see Materials and Methods) resulted in a significant decrease in wing blade aspect ratio. Lower and upper hinges correspond to the first and third quartiles, vertical lines extend to ± 1.5 times the interquartile range. Sample size is indicated for each RNAi line.

Knock-down experiments were conducted in groups, each including its own control (control_1-5). Statistical analysis was performed using one-way ANOVA, followed by Dunnett's multiple comparisons test. *$p \leq 0.05$, **$p \leq 0.01$, ***$p \leq 0.001$.

Appendix Fig S3. Ecd does not regulate mRNA levels and splicing of genes in general.

A Cartoon of late third-instar wing disc showing the expression pattern of *C765-Gal4* driver in light red.

B Knock-down of *ecd* in the whole wing disc using *C765-Gal4* (*C765>ecd^{RNAi}*). Ecd levels in control and *C765>ecd^{RNAi}* wing discs, analyzed by Western blot. The cell adhesion molecule *Drosophila* E-cadherin (DE-Cad) serves as a loading control. Ecd levels are markedly abolished upon *C765-Gal4*-driven *ecd^{RNAi}* expression.

C, D IF of control (C) and *C765>ecd^{RNAi}* (D) wing discs, stained for Cas3*. Loss of Ecd does not induce cell death in *C765>ecd^{RNAi}* wing discs, indicated by the absence of Cas3*-positive staining. Scale bars= 50 µm.

E-E'' Six transcripts of *eukaryotic initiation factor (elF)-4a* are annotated in the *Drosophila* genome. Gray boxes indicate 5' and 3' untranslated region (UTR). Exons are indicated by beige boxes, and introns by the black line (adapted from FlyBase). Red arrowheads mark the position of primers used to detect fully spliced cDNA, orange arrowheads mark the position of primers used to detect unspliced cDNA. (E') Representative result of semi-quantitative RT-PCR analysis for *eIF-4a* transcripts upon knock-down of *ecd* in the whole wing disc using *C765-GAL4* (*C765>ecd^{RNAi}*). Lanes for spliced *eIF-4a* mRNA are the same as shown in Fig EV2B', but here the cropped region extends to the height of unspliced *eIF-4a* mRNA as indicated by the higher running band amplified from DNA. (E'') Fold change in expression of spliced (red) and unspliced (orange) *eIF-4a* mRNA upon knock-down of *ecd* (relative to control wing discs). In both cases, transcript levels were not normalized. Error bars indicate ± SD, n=6. t-test, ns= not significant. Loss of Ecd does not change *eIF-4a* mRNA levels or

splicing. Thus, Ecd does not regulate mRNA levels and splicing of genes in general, consistent with previous results in the ring gland (Claudius et al, 2014). Rather, Ecd controls splicing of a specific subset of mRNAs in the wing disc, including those involved in glycolysis (see Fig EV2).

Appendix Fig S4. Knock-down of *brr2* or over-expression of *ecd*^{DN} mimics the effect of *ecd* knock-down on *Gapdh2* splicing

A, A' Spliced and unspliced mRNA levels of *Gapdh2* upon time-controlled knock-down of the spliceosomal component *brr2* in the whole wing disc (*C765*^{ts}*>brr2*^{RNAi}, see Appendix Fig S3A for the expression pattern of *C765-Gal4*). (A) Representative result of semi-quantitative RT-PCR analysis for *Gapdh2* transcripts upon knock-down of *brr2*. (A') Fold change in expression of spliced (red) and unspliced (orange) *Gapdh2* mRNA upon knock-down of *brr2* (relative to control wing discs). Error bars indicate ± SD, n=3. t-test, *p≤0.05, ***p≤0.001. Loss of Brr2 reduces the expression of spliced *Gapdh2* mRNA, but increases the expression of unspliced *Gapdh2* mRNA.

B, B' Spliced and unspliced mRNA levels of *Gapdh2* upon over-expression of *ecd* and *ecd*^{DN} in the whole wing disc (*C765>ecd*^{DN}, see Appendix Fig S3A for the expression pattern of *C765-Gal4*). (B) Representative result of semi-quantitative RT-PCR analysis for *Gapdh2* transcripts upon over-expression of full-length Ecd or over-expression of a C-terminally truncated form of Ecd in the whole wing disc (*C765>ecd* and *C765>ecd*^{DN}, respectively). The expression of the translation initiation factor *eIF-4a* serves as an internal normalization control. (B') Fold change in expression of spliced (red) and unspliced (orange) *Gapdh2* mRNA upon over-expression of *ecd* or over-expression of *ecd*^{DN} (relative to *C765>ecd* wing discs). In both cases, levels of *Gapdh2* transcripts were normalized to the levels of *eIF-4a*. Error bars indicate ± SD, n=4 (red, spliced), n=3 (orange, unspliced). t-test, ns= not significant, ***p≤0.001. Over-expression of *ecd*^{DN} increases the expression of unspliced *Gapdh2* mRNA.

Appendix Fig S5. Loss of Ecd in posterior Hh-producing cells does not induce Hh pathway activation in the anterior Hh-receiving cells

A-B''' Knock-down of *ecd* in the posterior compartment using *en-Gal4* (*en>ecd*^{RNAi}). (A, B) Cartoons of late third-instar wing disc showing the expression pattern of *en-Gal4* (B) driver in light red. (A'-B''') IF of control (A'-A''') and *en>ecd*^{RNAi} (B'-B''') wing discs, stained for Ecd::GFP (A', B'), Smo (A'', B''), and Ci_{155} (A''', B'''). Red dashed lines in B'-B''' mark the region of loss of Ecd::GFP expression. Next to the images in A'', A''' and B'', B''' are shown the quantifications of the respective stainings in the dorsal compartment of control (n=4) and *en>ecd*^{RNAi} (n=4) wing discs. Graphs show mean (thick line) ± SD (thin lines). Dashed yellow lines indicate the position of the A/P boundary. To compare stainings between control and *en>ecd*^{RNAi} wing discs, pixel intensity of control wing discs was adjusted to match pixel intensity of *en>ecd*^{RNAi} wing discs in the posterior compartment (mean ± SD as grey lines in B'' and B'''). Statistical analyses (t-test) revealed no significant difference of Smo or Ci_{155} expression between control and *en>ecd*^{RNAi} wing discs. Thus, loss of Ecd in the posterior compartment of *en>ecd*^{RNAi} wing discs does not cause Smo and Ci_{155} stabilization in the anterior compartment. Scale bars= 50 µm.

Appendix Fig S6. Loss of Ecd induces Hh pathway activation autonomously in the cells of the anterior compartment

A-B''' Knock-down of *ecd* in an anterior stripe along the A/P boundary using *dpp-Gal4, tub-Gal80*^{ts} (*dppGal*^{ts}*>ecd*^{RNAi}). (A, B) Cartoons of late third-instar wing disc showing the expression pattern of *dpp-Gal4* (B) driver in light red. (A'-B''') IF of control (A'-A''') and *dppGal*^{ts}*>ecd*^{RNAi} (B'-B''') wing discs, stained for Ecd::GFP (A', B'), Smo (A'', B''), and Ci_{155} (A''', B'''). Red dashed lines in B'-B''' mark the region of loss of Ecd::GFP expression. Next to the images in A'', A''' and B'', B''' are shown the quantifications of the respective stainings in the dorsal compartment of control (n=6) and *dppGal*^{ts}*>ecd*^{RNAi} (n=6) wing discs.

Graphs show mean (thick line) ± SD (thin lines). Dashed yellow lines indicate the position of the A/P boundary. To compare stainings between control and *dppGal^{ts}>ecd^{RNAi}* wing discs, pixel intensity of control wing discs was adjusted to match pixel intensity of *dppGal^{ts}>ecd^{RNAi}* wing discs in the posterior compartment (mean ± SD as grey lines in B'' and B'''). Statistical analyses (t-test) revealed significant differences of Smo ($p \leq 0.01$) and Ci_{155} ($p \leq 0.001$) expression between control and *dppGal^{ts}>ecd^{RNAi}* wing discs. Thus, loss of Ecd in anterior cells of *dppGal^{ts}>ecd^{RNAi}* wing discs is sufficient to increase Smo and Ci_{155} stabilization after 48 h of RNAi induction. Scale bars= 50 µm.

Appendix Table S1. Genotypes of wing discs and adult wings analyzed in this paper

**Figure**	**Label in figure**	**Genotype**
Fig 1	-ant. A or +ant. A	*ubi-AT1.03NL*
	control	*apGal*^{ts}*/+; +/ubi-AT1.03NL*
	apGal^{ts}*>Pfk*^{RNAi}	*+/UAS-dicer; apGal*^{ts}*/+; ubi-AT1.03NL/UAS-Pfk*^{RNAi} or *apGal*^{ts}*/+; ubi-AT1.03NL/UAS-Pfk*^{RNAi}
	apGal^{ts}*>Gapdh*^{RNAi}	*apGal*^{ts}*/+; UAS-Gapdh*^{RNAi}*/ubi-AT1.03NL*
	apGal^{ts}*>Glo1*^{RNAi}	*apGal*^{ts}*/UAS-Glo1*^{RNAi}*; +/ubi-AT1.03NL*
	apGal^{ts}*>ecd*^{RNAi}	*apGal*^{ts}*/UAS-ecd*^{RNAi}*; +/ubi-AT1.03NL*
Fig 2A, A'', D-E	control	*C765/+*
Fig EV2<br>Appendix Fig S3	*C765>ecd*^{RNAi}	*+/UAS-ecd*^{RNAi}*; C765/+*
Fig 2A', A'', E, F	*C765>Gapdh*^{RNAi}	*C765/UAS-Gapdh*^{RNAi}

Fig 2B-C’, E, F	*C765>ecd*	*C765/UAS-ecd*
Appendix Fig S4B-B’	*C765>ecd*^{DN}	*C765/UAS-ecd*^{DN}
Fig 3A-B’	control	*apGal*^{ts}*/+; fosEcd/+*
Fig 5A, B<br>Fig EV1D-H<br>Fig EV3	*apGal*^{ts}*>ecd*^{RNAi}	*apGal*^{ts}*/+; fosEcd/UAS-ecd*^{RNAi}
Fig 3C-C’<br>Fig EV5	*apGal*^{ts}*>Pfk*^{RNAi}	*+/UAS-dicer; apGal*^{ts}*/+; +/UAS-Pfk*^{RNAi}
Fig 3D-D’<br>Fig 6A/A’’’, C/C’’’	*apGal*^{ts}*>Gapdh*^{RNAi}	*apGal*^{ts}*/+; +/UAS-Gapdh*^{RNAi}
Fig 3E-E’	*apGal*^{ts}*>Glo1*^{RNAi}	*apGal*^{ts}*/UAS-Glo1*^{RNAi}

Fig 6C-J	control	*apGal*^{ts}*/dppZ; fosEcd/+*
	apGal^{ts}*>ecd*^{RNAi}	*apGal*^{ts}*/dppZ; fosEcd/UAS-ecd*^{RNAi}
	disp^{S037707}	*+/dppZ; disp*^{S037707}*/disp*^{S037707}*, UAS-ecd*^{RNAi}
	disp^{S037707}<br>*apGal*^{ts}*>ecd*^{RNAi}	*apGal*^{ts}*/dppZ; disp*^{S037707}*/disp*^{S037707}*, UAS-ecd*^{RNAi}
Fig 6A-A'', C-C''	control	*apGal*^{ts}*/+*
	apGal^{ts}*>ecd*^{RNAi}	*apGal*^{ts}*/+; +/UAS-ecd*^{RNAi}
Fig 6D-E''	-gA or +gA	Oregon-R-C
Fig EV1	control	*bcg/UAS-CD8::GFP; +/UAS-CD8::GFP*
	apGal^{ts}*>CD8::GFP*	*apGal*^{ts}*/UAS-CD8::GFP; +/UAS-CD8::GFP*
Fig EV4	*apGal*^{ts}*>ecd*	*apGal*^{ts}*/+;+/UAS-ecd*
	apGal^{ts}*>ecd*^{DN}	*apGal*^{ts}*/+;+/UAS-ecd*^{DN}
	apGal^{ts}*>brr2*^{RNAi}	*apGal*^{ts}*/UAS-brr2*^{RNAi}

Appendix Fig S1A, B	control	apGal^{ts}/Gapdh1::GFP^{V5}
	apGal^{ts}>Gapdh^{RNAi}	apGal^{ts}/Gapdh1::GFP^{V5}; +/UAS-Gapdh^{RNAi}
Appendix Fig S1C, D	control	GFP^{Myc}::Gapdh2/+; apGal^{ts}/+
	apGal^{ts}>Gapdh^{RNAi}	GFP^{Myc}::Gapdh2/+; apGal^{ts}/+; +/UAS-Gapdh^{RNAi}
Appendix Fig S2	control_1-5	C765/+
	C765>Eno^{RNAi}	+/UAS-Eno^{RNAi}; C765/+
	C765>Treh^{RNAi}	+/UAS-Treh^{RNAi}; C765/+ or C765/UAS-Treh^{RNAi}
	C765>Ald^{RNAi}	+/UAS-Ald^{RNAi};; C765/+ or +/UAS-Ald^{RNAi}; C765/+
	C765>Gapdh1^{RNAi}	+/UAS-Gapdh1^{RNAi}; C765/+ or C765/UAS-Gapdh1^{RNAi}
	C765>Gapdh2^{RNAi}	+/UAS-Gapdh2^{RNAi}; C765/+ or C765/UAS-Gapdh2^{RNAi}
	C765>Pgk^{RNAi}	+/UAS-Pgk^{RNAi}; C765/+ or C765/UAS-Pgk^{RNAi}
	C765>PyK^{RNAi}	+/UAS-PyK^{RNAi}; C765/+ or C765/UAS-PyK^{RNAi}
	C765>Pfk^{RNAi}	C765/UAS-Pfk^{RNAi}
	C765>Glo1^{RNA}	+/UAS-Glo1^{RNAi}; C765/+
	C765>Hex-A^{RNAi}	C765/UAS-Hex-A^{RNAi}
Appendix Fig S4A-A’	C765^{ts}>brr2^{RNAi}	+/UAS-brr2^{RNAi}; C765^{ts}/+

Appendix Fig S5	control	*fosEcd*
	en>ecd^{RNAi}	*en(105)-Gal4/ UAS-ecd*^{RNAi}*; fosEcd/+*
Appendix Fig S6	control	*dppGal*^{ts}*, fosEcd/+*
	dppGal^{ts}*>ecd*^{RNAi}	*dppGal*^{ts}*, fosEcd/UAS-ecd*^{RNAi}

Appendix Table S2. Oligonucleotides used for the generation of transgenic flies

Oligonucleotide	Sequence
ubi-AT1.03NL	
AT_NL_AvrII_f	CATTAGCCTAGGCTAGAACTAGTGGATCCGAGCTCGAGTATGGTGAGC
AT_NL_SpeI_r	TAGCACTAGTTTACTCGATGTTGTGGCGGATC
Gapdh1::GFP and ***Gapdh2::GFP***	
Gapdh1_sense	GTCGAACTCAGCCATGTCGAAGAT
Gapdh1_antisense	AAACATCTTCGACATGGCTGAGTT
Gapdh1_left_f	GCTGCAGGAATTCGATATCAGCACATAGCTCATACCGTAC
Gapdh1_left_r	TGGACACCATGGCTGAGTTCCTGCTGTC
Gapdh1_right_f	GGATAGCACCTCGAAGATCGGAATTAACG
Gapdh1_right_r	AGGGAACAAAAGCTGGGTACAGCAACGTTCTTCATTCG
Gapdh1_sGFP_f	GAACTCAGCCATGGTGTCCAAGGGCGAG
Gapdh1_sGFP_r	CGATCTTCGAGGTGCTATCCAGGCCCAG
Gapdh2_sense	GTCGAAGTTAACCATGTCGAAGAT
Gapdh2_antisense	AAACATCTTCGACATGGTTAACTT

NotI_Gapdh2_f	ATAAGAATGCGGCCGCCGTGACTCAAAAGGTCACATTC
NcoI_Gapdh2_r	TCCTCGCCCTTGGACACCATGGTTAACTTTTTGTTTATCT
EarI_Gapdh2_f	ACAGAAGTTGATTTCCGAAGAGGACTTGGAGCAGAAACTGATCTCGGAGGAGGATCTCTCGAAGATTGGT ATCAATGGA
KpnI_Gapdh2_r	CGGGGTACCAACTTTTACGTAGAGTGTGGGTGG
NcoI_Gapdh2_sGFP_f	AGATAAACAAAAAGTTAACCATGGTGTCCAAGGGCGAGGA
EarI_Gapdh2_sGFP_r	AGTCCTCTTCGGAAATCAACTTCTGTTCCAGATCTTCCTCGCTGATGAGCTTTTGCTCCTCGAGCTTGTACA GCTCATC
UAS-ecd and ***UAS-ecd***$^{\boldsymbol{DN}}$	
NotI_cEcd_f	AAAGCGGCCGCCAAAGGATGAGCAAGATTCCAGGC
SacI_cEcd_r	AGAAGCTGTGCAGCGCTATG
SacI_fosEcd_f	CGCACTTCTCCATTCATCGCCAGG
KpnI_fosEcd_r	AAAGGTACCTTACTTGTCGTCGTCATCCTTGTAGT
Q650*_r	GATTGGTATGCACTTCCGATTGGTAGCTATCCATC
Q650*_f	GATGGATAGCTACCAATCGGAAGTGCATACCAATC

Appendix Table S3. Oligonucleotides used for semi-quantitative PCR

Oligonucleotide	Sequence
eIF4A-F	TATGGTTTCGAGAAGCCGTC
eIF4A-R	CCTGCTTCACGTTGACGTAA
eIF4A-intron-F	TCCTGTTTTGTGTTCTGGCA
eIF4A-intron-R	CTCGTGTCGATTTGCTGAAG
Gapdh2-F	CAGCCATCACAGTCGATTCC
Gapdh2-R	GGACTCCACGATGTATTCGG
Gapdh2-intron-F	CCTCAATTCCGCGGAAAACTTTCCA
Gapdh2-intron-R	CCGGGTCGCGTTCGCTGAAG
Gapdh2-intron-R2	GTCGTTGATCACCTTGGCCAGAGGA
Pdk-F	AAATATAGCCAACATGCGCC
Pdk-R	TGCGAGACATGTAGAGCCTG
Pdk-intron-F	ATAAAAGCGAAGGTGAGCGA
Pdk-intron-R	TGGGTTCAAGAAACTGAGGC
PyK-F	TCTGGGTATTGAGATTCCCG
PyK-R	TACTTCCTGAGCGGCAGAAT
PyK-intron-F	GGTTTTTGTTTCTCCAGGTCG
smo-F	CGCGCATGCCAAACATTAT
smo-R	CACGTGCTTCAGGGCATAGT
smo-intron-F	ATGGTGTGGGCAATTAAAATAACTT
smo-intron-R	GCATAGTAGTCGTTCAGCTTATCGT
