# Supplementary material for: Glycolysis regulates Hedgehog signalling via the plasma membrane potential
Source: EMBO J. 2020 Oct 6;39(21):e101767. doi: 10.15252/embj.2019101767 (PMC7604625; doi:10.15252/embj.2019101767)
Supplement: Supplementary file 2 — Expanded View Figures PDF [file EMBJ-39-e101767-s002.pdf]

Expanded View Figures

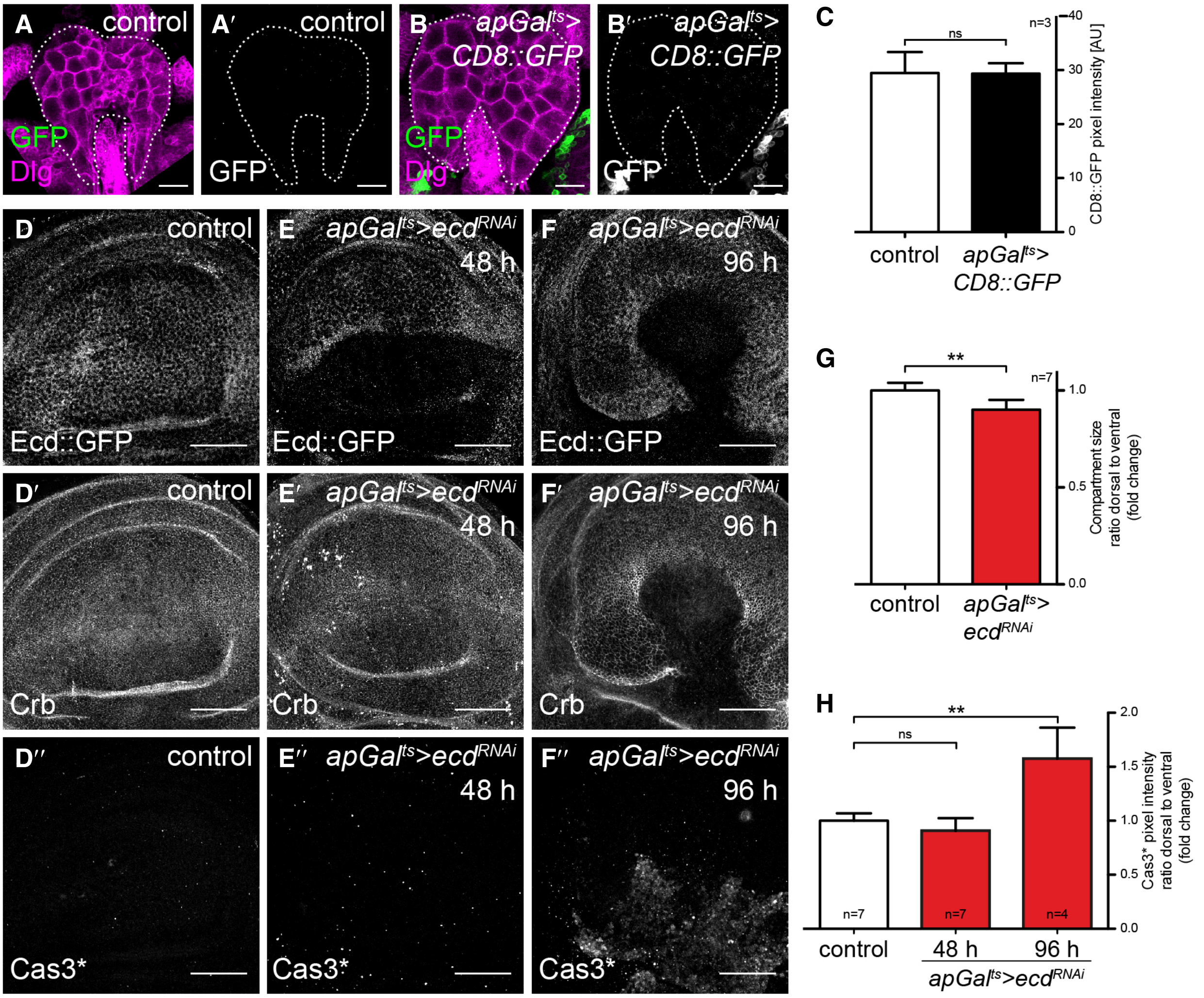

Figure EV1. Loss of Ecdysoneless reduces tissue growth.

A–B′ Over-expression of membrane-targeted CD8::GFP using *apGal^{ts}*. IF of control (A, A′) and *apGal^{ts}>CD8::GFP* ring glands (B, B′), stained for GFP (green in A, B, grey scale in A′, B′) and the lateral membrane marker Discs-large (Dlg, magenta in A, B). The white dashed line outlines the periphery of the ring gland. Scale bars = 25 μm.

C Quantification of CD8::GFP signal intensity in control and *ap>CD8::GFP* ring glands. For each condition, 3 ring glands were quantified. The *apGal^{ts}* driver does not drive expression in the steroid hormone-producing ring gland. Error bars indicate ± SD. *t*-test, ns = not significant.

D–F″ Time-controlled knock-down of *ecd* in the dorsal compartment using *apGal^{ts}>ecd^{RNAi}* (see Fig 1C for the expression pattern of *apGal^{ts}*). Knock-down of *ecd* is shown by the loss of fosmid-encoded Ecd (Ecd::GFP) expression. IF of control (D-D″) and *apGal^{ts}>ecd^{RNAi}* (48 h: E-E″, 96 h: F-F″) wing discs, stained for Ecd::GFP (D-F), the apical membrane marker Crumbs (Crb, D′-F′) and activated Caspase-3 (Cas3*, D″-F″). Lack of Ecd function induces cell death, indicated by Cas3*-positive staining and loss of Crb after 96 h of RNAi induction. Scale bars = 50 μm.

G Quantification of the size of the dorsal to ventral compartment in control and *apGal^{ts}>ecd^{RNAi}* wing discs after 48 h of RNAi induction, shown as fold change relative to control. Loss of Ecd reduces the size of the dorsal compartment in *apGal^{ts}>ecd^{RNAi}* wing discs. For each condition, dorsal/ventral compartment size was quantified for 7 wing discs. Error bars indicate ± SD. *t*-test, $^{**}P \leq 0.01$.

H Quantification of Cas3* signal intensity in the dorsal to ventral compartment in control (n = 7) and *apGal^{ts}>ecd^{RNAi}* wing discs at 48 h (n = 7) and 96 h (n = 4) of RNAi induction, shown as a fold change from control. Loss of Ecd induces cell death after long-term RNAi induction. Error bars indicate ± SD. *t*-test, ns = not significant, $^{**}P \leq 0.01$.

Source data are available online for this figure.

 © 2020 The Authors

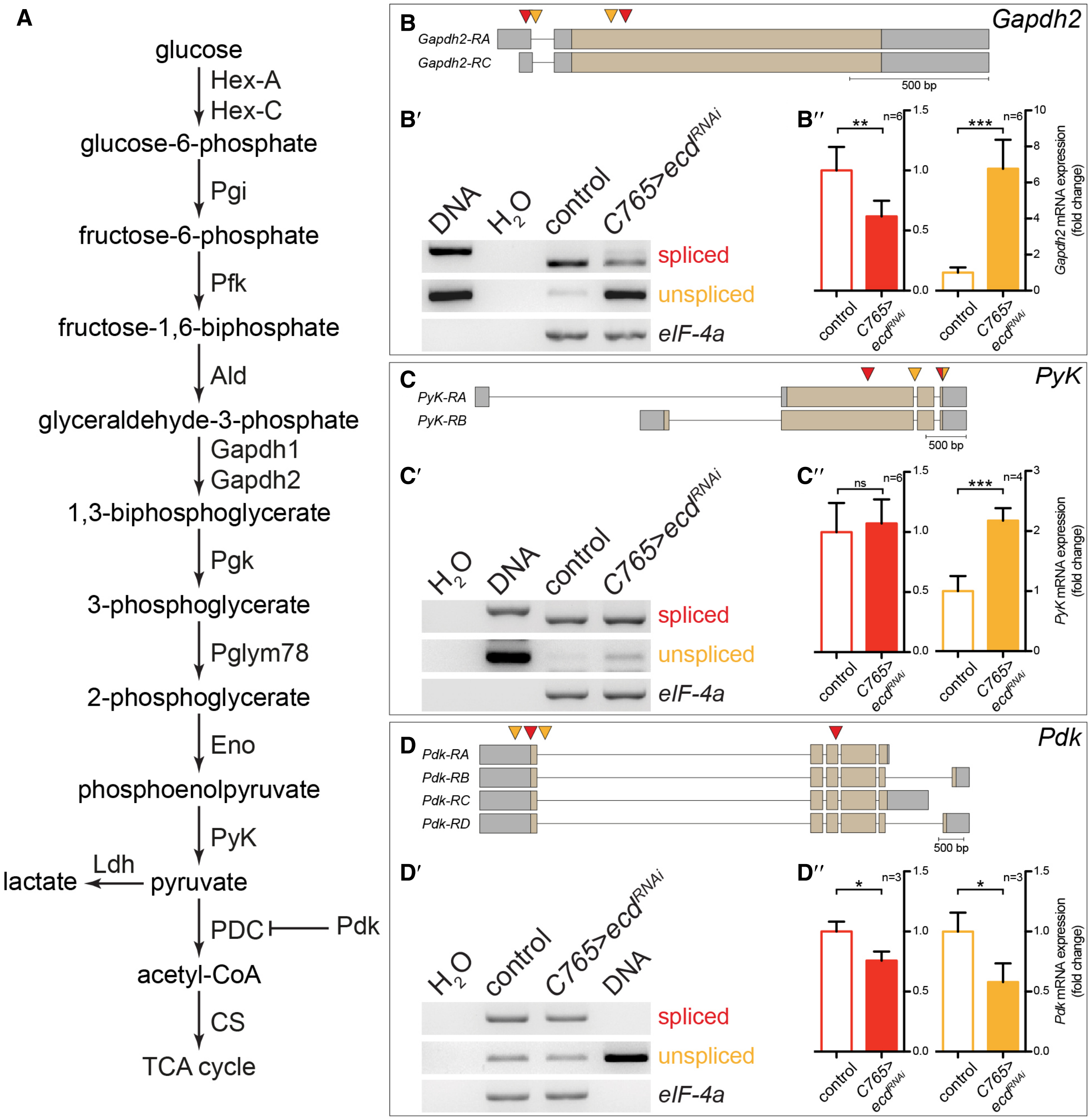
A
glucose
Hex-A
Hex-C
glucose-6-phosphate
Pgi
fructose-6-phosphate
Pfk
fructose-1,6-biphosphate
Ald
glyceraldehyde-3-phosphate
Gapdh1
Gapdh2
1,3-biphosphoglycerate
Pgk
3-phosphoglycerate
Pglym78
2-phosphoglycerate
Eno
phosphoenolpyruvate
PyK
lactate
Ldh
pyruvate
PDC
Pdk
acetyl-CoA
CS
TCA cycle
B
Gapdh2
Gapdh2-RA
Gapdh2-RC
500 bp
B′
DNA
H2O
control
C765>ecdRNAi
spliced
unspliced
eIF-4a
B″
**
n=6
***
n=6
Gapdh2 mRNA expression (fold change)
control
C765>ecdRNAi
C
PyK
PyK-RA
PyK-RB
500 bp
C′
H2O
DNA
control
C765>ecdRNAi
spliced
unspliced
eIF-4a
C″
ns
n=6
***
n=4
PyK mRNA expression (fold change)
control
C765>ecdRNAi
D
Pdk
Pdk-RA
Pdk-RB
Pdk-RC
Pdk-RD
500 bp
D′
H2O
control
C765>ecdRNAi
DNA
spliced
unspliced
eIF-4a
D″
*
n=3
*
n=3
Pdk mRNA expression (fold change)
control
C765>ecdRNAi

Figure EV2.

Figure EV2. Ecd regulates mRNA levels and splicing of genes involved in carbohydrate metabolism.

A Overview of the glycolytic pathway and the entry into the tricarboxylic acid (TCA) cycle. *Drosophila* gene symbols of enzymes are given. PDC stands for pyruvate dehydrogenase complex.

B–B″ Two *Glyceraldehyde-3-phosphate dehydrogenase 2* (*Gapdh2*) transcripts are annotated in the *Drosophila* genome. Grey boxes indicate 5′ and 3′ untranslated regions (UTR). Exons are indicated by beige boxes and introns by the black line (adapted from FlyBase). Red arrowheads mark the position of primers used to detect fully spliced cDNA; orange arrowheads mark the position of primers used to detect unspliced cDNA. (B′) Representative result of semi-quantitative RT–PCR analysis of *Gapdh2* transcripts upon knock-down of *ecd* in the whole wing disc using *C765-GAL4* (*C765>ecd^{RNAi}*, see Appendix Fig S3A for the expression pattern of *C765-GAL4*). The expression of the translation initiation factor *eIF-4a* serves as an internal normalization control. (B″) Levels of spliced (red) and unspliced (orange) *Gapdh2* mRNAs upon knock-down of *ecd*, shown as a fold change relative to control. Levels of *Gapdh2* transcripts were normalized to the levels of *eIF-4a*. Error bars indicate ± SD, n = 6. t-test, **$P \leq 0.01$, ***$P \leq 0.001$. Loss of Ecd reduces the levels of spliced *Gapdh2* mRNA but increases the levels of unspliced *Gapdh2* mRNA.

C–C″ Two *Pyruvate kinase* (*PyK*) transcripts are annotated in the *Drosophila* genome (adapted from FlyBase). (C′) Representative result of semi-quantitative RT–PCR analysis of *PyK* transcripts upon knock-down of *ecd*. (C″). Levels of spliced (red) and unspliced (orange) *PyK* mRNAs upon knock-down of *ecd*, shown as a fold change relative to control. Levels of *PyK* transcripts were normalized to the levels of *eIF-4a*. Error bars indicate ± SD, n = 6 (red, spliced), n = 4 (orange, unspliced). t-test, ns = not significant, ***$P \leq 0.001$. Loss of Ecd increases unspliced *PyK* mRNA.

D–D″ Four *Pyruvate dehydrogenase kinase* (*Pdk*) transcripts are annotated in the *Drosophila* genome (adapted from FlyBase). (D′) Representative result of semi-quantitative RT–PCR analysis for *Pdk* transcripts upon knock-down of *ecd*. (D″). Levels of spliced (red) and unspliced (orange) *Pdk* mRNAs upon knock-down of *ecd*, shown as a fold change relative to control. Levels of *Pdk* transcripts were normalized to the levels of *eIF-4a*. Error bars indicate ± SD, n = 3. t-test, *$P \leq 0.05$. Loss of Ecd reduces *Pdk* mRNA levels, consistent with results from mammalian cell culture (Dey *et al*, 2012).

Source data are available online for this figure.

 © 2020 The Authors

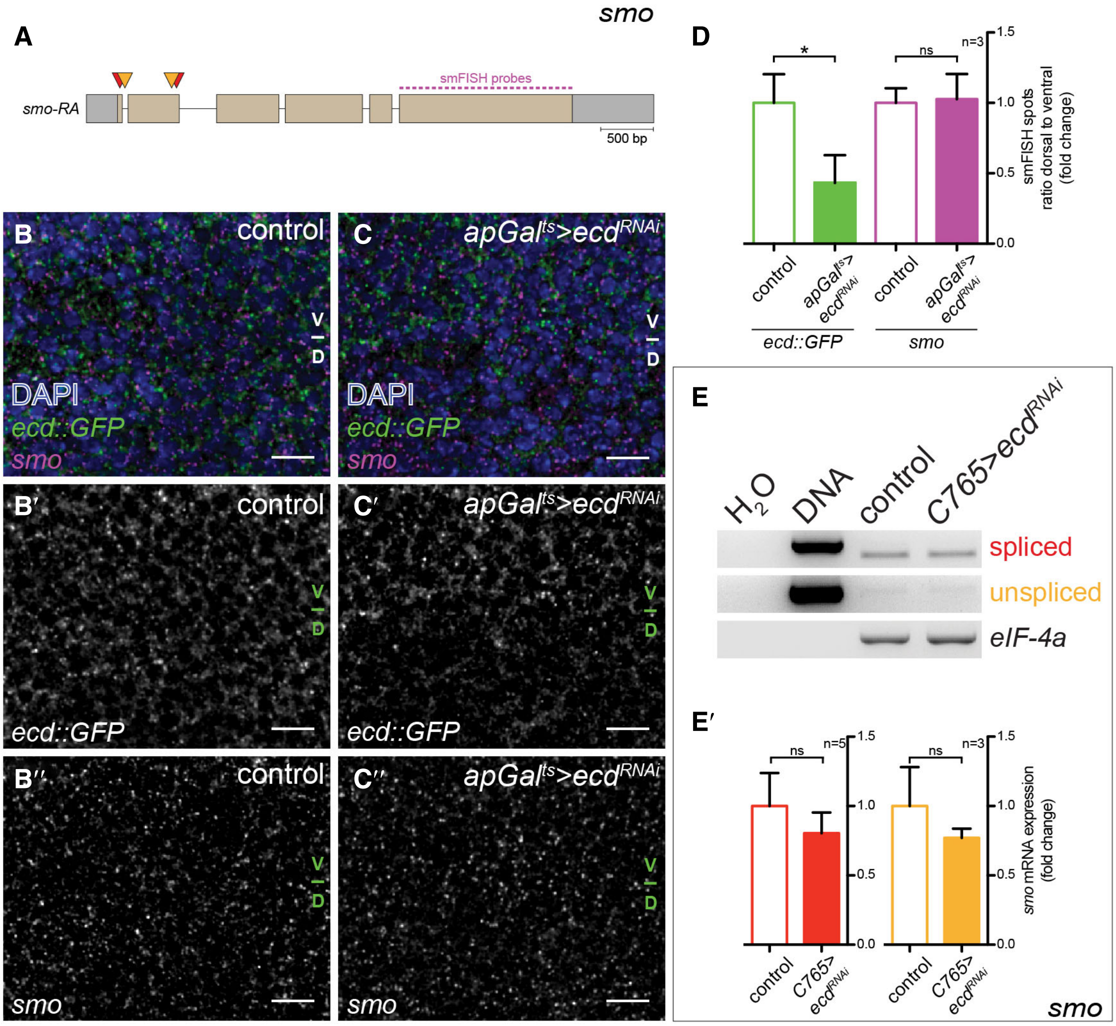

Figure EV3. Loss of Ecd does not alter mRNA levels or splicing of *smo*.

A One *smo* transcript is annotated in the *Drosophila* genome. Grey boxes indicate 5′ and 3′ untranslated region (UTR). Exons are indicated by beige boxes and introns by the black line (adapted from FlyBase). Red arrowheads mark the position of primers used to detect fully spliced cDNA; orange arrowheads mark the position of primers used to detect unspliced cDNA. Dashed pink line represents the target sequence against which the single-molecule fluorescence *in situ* hybridization (smFISH) probes were designed.

B–C″ Time-controlled knock-down of *ecd* in the dorsal compartment of the wing discs (see Fig 1C for the expression pattern of *apGal*^{ts}). Dual-colour smFISH with probes against *ecd::GFP* (green in B, C, grey scale in B′, C′) and *smo* (magenta in B, C, grey scale in B″, C″) mRNA in control (B-B″) and *apGal*^{ts}>*ecd*^{RNAi} (C-C″) wing discs after 48 h of RNAi induction. DAPI staining was used to visualize cell nuclei. V/D indicates the boundary between the ventral and dorsal compartments. Scale bars = 10 μm.

D Quantification of the ratio of *ecd::GFP* (green) and *smo* (magenta) mRNA levels in the dorsal to ventral compartment of control and *apGal*^{ts}>*ecd*^{RNAi} wing discs, shown as fold change relative to control. For each condition, dorsal/ventral signal intensities of smFISH probes were quantified for 3 wings discs. Error bars indicate ± SD. *t*-test, ns = not significant, **P* < 0.05. Knock-down of *ecd*, indicated by the significant reduction of *ecd::GFP* mRNA levels does not alter the expression of *smo* mRNA.

E, E′ Semi-quantitative RT–PCR analysis for *smo* transcripts upon knock-down of *ecd* in the whole wing disc (*C765*>*ecd*^{RNAi}, see Appendix Fig S3A for the expression pattern of *C765-Gal4*). The expression of the translation initiation factor *eIF-4a* serves as an internal normalization control. Loss of Ecd does not affect *smo* mRNA expression. (E′). Levels of spliced (red) and unspliced (orange) *smo* mRNA upon knock-down of *ecd*, shown as a fold change relative to control. Levels of *smo* transcripts were normalized to the levels of *eIF-4a*. Error bars indicate ± SD, *n* = 5 (red, spliced), *n* = 3 (orange, unspliced). *t*-test, ns = not significant.

Source data are available online for this figure.

© 2020 The Authors

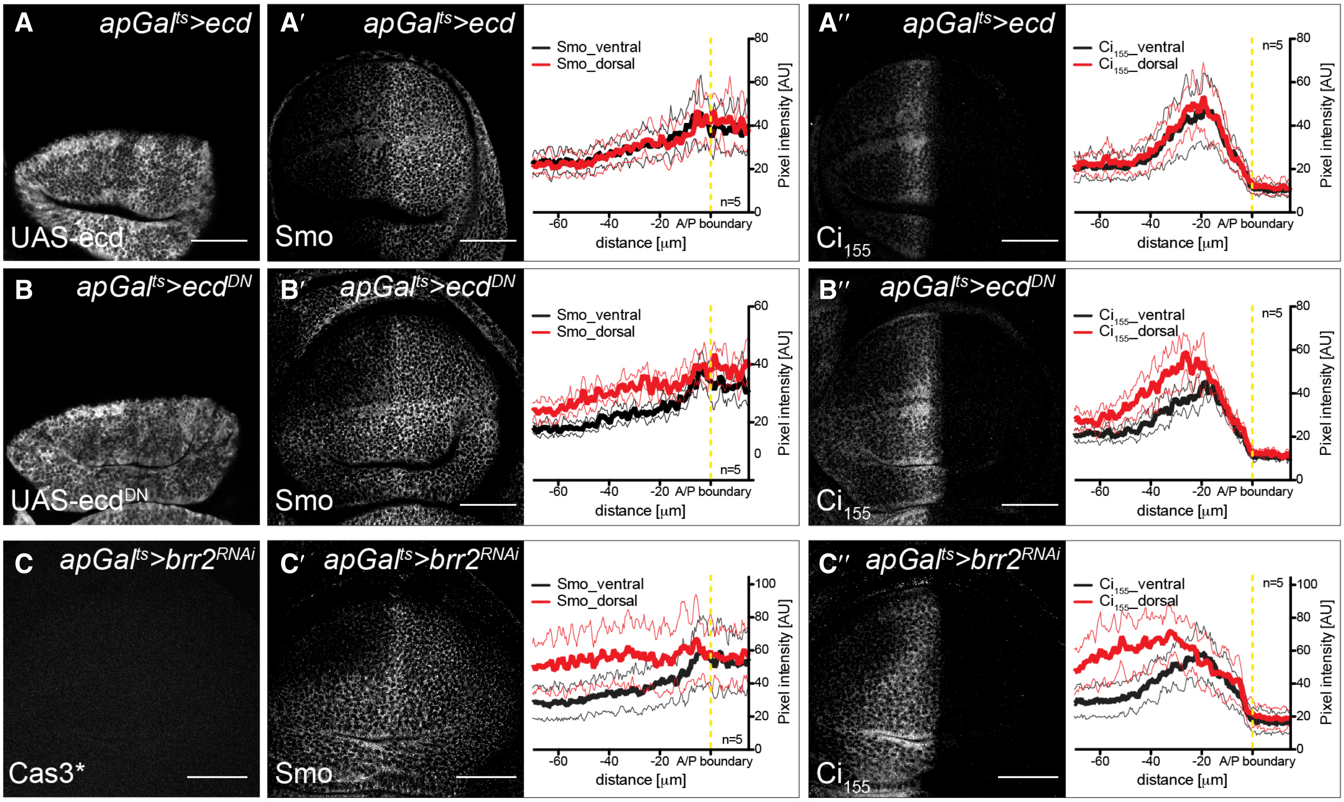

Figure EV4. Over-expression of *ecd*^{DN} or knock-down of *brr2* mimics the effect of *ecd* knock-down on stabilization of Smo and Ci_{155}.

A–B″ Over-expression of full-length Ecd (*apGal^{ts}>ecd*) and C-terminally truncated form of Ecd (*apGal^{ts}>ecd^{DN}*) in the dorsal compartment of the wing disc (see Fig 1C for the expression pattern of *apGal^{ts}*). IF of *apGal^{ts}>ecd* (A-A″) and *apGal^{ts}>ecd^{DN}* (B-B″) wing discs, stained for the respective over-expression constructs (A, B), Smo (A′, B′) or Ci_{155} (A″, B″). Next to the images are quantifications of the respective stainings in the dorsal versus ventral compartments of *apGal^{ts}>ecd* and *apGal^{ts}>ecd^{DN}* wing discs (n = 5). Graphs show mean (thick line) ± SD (thin lines). Dashed yellow lines indicate the position of the A/P boundary. Statistical analyses (t-test) revealed significant differences in Smo and Ci_{155} expression in the dorsal compartment between *apGal^{ts}>ecd* and *apGal^{ts}>ecd^{DN}* (Smo: $P \leq 0.01$, Ci_{155}: $P \leq 0.05$) wing discs. Thus, over-expression of *ecd^{DN}* leads to the stabilization of Smo and Ci_{155}. Wing discs were analysed after 96 h of *ecd* or *ecd^{DN}* over-expression. Scale bars = 50 µm.

C–C″ Time-controlled knock-down of *brr2* in the dorsal compartment of the wing disc (*apGal^{ts}>brr2^{RNAi}*, see Fig 1C for the expression pattern of *apGal^{ts}*). IF of *apGal^{ts}>brr2^{RNAi}* wing discs after 30 h of RNAi induction, stained for Cas3* (C), Smo (C′) or Ci_{155} (C″). Next to the images are quantifications of the respective stainings in the dorsal versus ventral compartments (n = 5). Graphs show mean (thick line) ± SD (thin lines). Dashed yellow lines indicate the position of the A/P boundary. Statistical analyses (t-test) revealed significant differences in Smo and Ci_{155} expression in the dorsal compartment between control and *apGal^{ts}>brr2^{RNAi}* ($P \leq 0.001$) wing discs. Thus, loss of Brr2 leads to the stabilization of Smo and Ci_{155}. Scale bars = 50 µm.

Source data are available online for this figure.

© 2020 The Authors

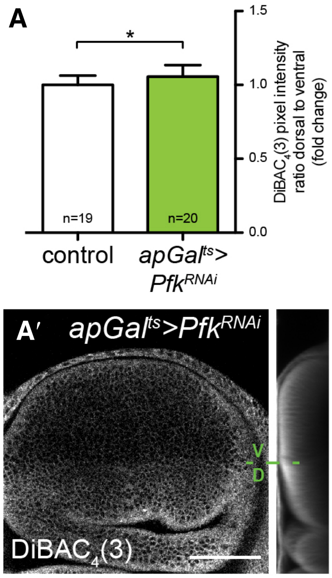

Figure EV5. Loss of Pfk depolarizes the plasma membrane.

A–A′ Time-controlled knock-down of *Pfk* in the dorsal compartment of the wing disc (see Fig 1C for the expression pattern of *apGal*^{ts}) and measurement of the plasma membrane potential using DiBAC$_4$(3). (A) Plasma membrane potential quantified as the ratio of DiBAC$_4$(3) signal intensity in the dorsal to ventral compartment in control (n = 19) and *apGal*^{ts}>*Pfk*^{RNAi} (n = 20) wing discs, shown as a fold change relative to control. Error bars indicate ± SD. *t*-test, *$P \leq 0.05$. (A′-A‴) Representative image of DiBAC$_4$(3) assay in *apGal*^{ts}>*Pfk*^{RNAi} (A′) wing discs 120 h after RNAi induction. V/D indicates the boundary between the ventral and dorsal compartments. A sum projection of cross-sections along the A/P axis is shown next to the maximal projection. Loss of Pfk alters the plasma membrane potential. Scale bar = 50 µm.

Source data are available online for this figure.

© 2020 The Authors
