# Supplementary material for: Glycolysis regulates Hedgehog signalling via the plasma membrane potential
Source: EMBO J. 2020 Oct 6;39(21):e101767. doi: 10.15252/embj.2019101767 (PMC7604625; doi:10.15252/embj.2019101767)
Supplement: Supplementary file 3 — Code EV1 [file EMBJ-39-e101767-s003.zip › Program_for_final_submission/ FRET_Read_me.docx]

**Steps to use the MATLAB script for estimating metabolite FRET**

1. Prepare the data for analysis by separately saving the CFP and YFP files with different filenames
2. Input the path of the folder containing the CFP and YFP files in the line 9 showing “read_folder”
3. Input the file pattern to identify CFP and YFP files in lines 11 and 12 of the MATLAB code
4. Input the number of samples in line 17
5. Run the code
6. Draw the background rectangle in the first window that opens
7. Draw freehand ROI to select the region of interest in the next window
8. The program will repeat steps 6 and 7 till ROIs are selected in all the samples
9. The Program automatically saves the .mat files containing the FRET and total donor intensity matrices.
10. Double click the “All_Disc_mean” variable in the workspace of MATLAB and you can see the average FRET values of all the samples. You can copy this to any worksheet like excel for further analysis.
