# Supplementary material for: Glycolysis regulates Hedgehog signalling via the plasma membrane potential
Source: EMBO J. 2020 Oct 6;39(21):e101767. doi: 10.15252/embj.2019101767 (PMC7604625; doi:10.15252/embj.2019101767)
Supplement: Supplementary file 3 — Code EV1 [file EMBJ-39-e101767-s003.zip › Program_for_final_submission/Metabolite_Sensor_FRET_Annotated_document.pdf]

This MATLAB program estimates the FRET efficiency in the wing disc at each Z-plane from a sensitized emission method of FRET.

Clearing memory and Command terminal

```
clc;
clear;
```

Selecting input the directory and file path

```
read_folder = '-Add folder path here-';
cd(read_folder);
YFP_files = dir('C2*.tif');                    Pattern for YFP files
CFP_files = dir('C1*.tif');                    Pattern for CFP files
```

Drawing ROIs for all the discs

```
F = 2;                                         Enter the number of Discs
back_rect_array = [];                          Initialize background rectangle array
mask_array = [];                               Initialize mask array
file_inf = imfinfo(CFP_files(1).name);
N = numel(file_inf); %                         Estimate the number of Z slices
[row,col] = size(CFP_files(1).name);           Estimate image size

for d=1:F
        test = imread(CFP_files(d).name,1);
        [row,col] = size(test);
        CFP = uint16(zeros(row,col,N));YFP = uint16(zeros(row,col,N));
        for i=1:N
            CFP(:,:,i) = imread(CFP_files(d).name,i);      Generate 3-D array for CFP
            YFP(:,:,i) = imread(YFP_files(d).name,i);      Generate 3-D array for YFP
        end

        YFP_max = mean(YFP,3);                 Generate mean projected image for YFP
        N = numel(CFP(1,1,:));
        H = fspecial('average',[5,5]);         Creating an averaging kernel

        [I_crop,back_rect] = imcrop(YFP_max,[]);       Get background rectangle
        imshow(YFP_max,[]);
        h = imfreehand();  %                   Draw a freehand ROI
        mask = h.createMask; %                 Convert freehand ROI into mask
        back_rect_array = cat(1,back_rect_array,back_rect); Generate array of Background ROI
        mask_array = cat(3,mask_array,mask);   Generate an array of masks
end
```

Computing FRET the wing discs in 3-Dimension for all the discs selected

```
All_disc_mean = [];
count =1;
for d=1:F

        for i=1:N;
            CFP(:,:,i) = imread(CFP_files(d).name,i);      Generate a 3-D array for CFP
            YFP(:,:,i) = imread(YFP_files(d).name,i);      Generate a 3-D array for YFP
        end
            H = fspecial('average',[5,5]);                 Generate a 5X5 smoothing kernel
```

```
    back_rect1 = back_rect_array(count,:);          Select a background ROI from array
    mask1 = mask_array(:,:,count);                  Select a mask from array of masks

    FRET_Z = zeros(row,col,N);Tot_donor_Z = zeros(row,col,N);
    for j=1:N
        back_cfp = imcrop(CFP(:,:,j),back_rect1);   Generate background image for CFP
        back_yfp = imcrop(YFP(:,:,j),back_rect1);   Generate background image for YFP
        mn1 = mean(mean(back_cfp));                 Compute mean background for CFP
        mn2 = mean(mean(back_yfp));                 Compute mean background for YFP
        cfp_filt = imfilter(CFP(:,:,j),H); %.       5X5 smoothing of the image for CFP
        yfp_filt = imfilter(YFP(:,:,j),H); %.       5X5 smoothing of the image for CFP
        cfp_back_sub = double(cfp_filt - mn1);      Subtract background from CFP image
        yfp_back_sub = double(yfp_filt - mn2);      Subtract background from CFP image
        leak = 0.4*cfp_back_sub;                    Computing leakage
        PFRET = yfp_back_sub - leak;                Get FRET intensity
        tot_donor = PFRET + cfp_back_sub;           Get Total donor intensity
        FRET = PFRET./tot_donor;                    Estimate FRET Efficiency
        FRET_Z(:,:,j) = FRET;
        Tot_donor_Z(:,:,j) = tot_donor.*mask1;      Get masked total donor intensity
    end

    Tot_don_lin = Tot_donor_Z(Tot_donor_Z>0);
    mn = mean(Tot_don_lin);                         Get mean intensity of Total Donor
    Generate background by rolling ball method
    tot_don_back = imopen(Tot_donor_Z,strel('disk',25));
    Tot_don_backsub = Tot_donor_Z - tot_don_back;   Subtract Background
    Tot_don_filt = medfilt3(Tot_don_backsub);
    Tot_don_thresh = (Tot_don_filt>0.20*mn);        Threshold the total donor


    Thresholding FRET signal
    FRET_thresh = zeros(row,col,N);
    for k=1:N
        FRET_thresh(:,:,k) = FRET_Z(:,:,k).*Tot_don_thresh(:,:,k);
    end

  Saving FRET values as a MATLAB matrix
  filename = strcat('ATP_','Disc_',num2str(d,'%03d'),'.mat');
  save(filename, 'FRET_thresh','Tot_donor_Z');      Save FRET and total donor matrix
  count = count+1
  FRET_lin = FRET_thresh(FRET_thresh>0);
  mn_FRET = mean(FRET_lin);
  All_disc_mean = cat(1,All_disc_mean,mn_FRET);

end
```
