# Supplementary material for: Glycolysis regulates Hedgehog signalling via the plasma membrane potential
Source: EMBO J. 2020 Oct 6;39(21):e101767. doi: 10.15252/embj.2019101767 (PMC7604625; doi:10.15252/embj.2019101767)
Supplement: Supplementary file 3 — Code EV1 [file EMBJ-39-e101767-s003.zip › Program_for_final_submission/Metabolite_Sensor_FRET_document.docx]

**This MATLAB program estimates the FRET efficiency in the wing disc at each Z-plane from a sensitized emission method of FRET.**

**Clearing memory and Command terminal**

clc;

clear;

**Selecting input the directory and file path**

read_folder = '-Add folder path here-';

cd(read_folder);

YFP_files = dir('C2*.tif'); Pattern for YFP files

CFP_files = dir('C1*.tif'); Pattern for CFP files

**Drawing ROIs for all the discs**

F = 2; Enter the number of Discs

back_rect_array = []; Initialize background rectangle array

mask_array = []; Initialize mask array

file_inf = imfinfo(CFP_files(1).name);

N = numel(file_inf); % Estimate the number of Z slices

[row,col] = size(CFP_files(1).name); Estimate image size

for d=1:F

test = imread(CFP_files(d).name,1);

[row,col] = size(test);

CFP = uint16(zeros(row,col,N));YFP = uint16(zeros(row,col,N));

for i=1:N

CFP(:,:,i) = imread(CFP_files(d).name,i); Generate 3-D array for CFP

YFP(:,:,i) = imread(YFP_files(d).name,i); Generate 3-D array for YFP

end

YFP_max = mean(YFP,3); Generate mean projected image for YFP

N = numel(CFP(1,1,:));

H = fspecial('average',[5,5]); Creating an averaging kernel

[I_crop,back_rect] = imcrop(YFP_max,[]); Get background rectangle

imshow(YFP_max,[]);

h = imfreehand(); % Draw a freehand ROI

mask = h.createMask; % Convert freehand ROI into mask

back_rect_array = cat(1,back_rect_array,back_rect); Generate array of Background ROI

mask_array = cat(3,mask_array,mask); Generate an array of masks

end

**Computing FRET the wing discs in 3-Dimension for all the discs selected**

All_disc_mean = [];

count =1;

for d=1:F

for i=1:N;

CFP(:,:,i) = imread(CFP_files(d).name,i); Generate a 3-D array for CFP

YFP(:,:,i) = imread(YFP_files(d).name,i); Generate a 3-D array for YFP

end

H = fspecial('average',[5,5]); Generate a 5X5 smoothing kernel

back_rect1 = back_rect_array(count,:); Select a background ROI from array

mask1 = mask_array(:,:,count); Select a mask from array of masks

FRET_Z = zeros(row,col,N);Tot_donor_Z = zeros(row,col,N);

for j=1:N

back_cfp = imcrop(CFP(:,:,j),back_rect1); Generate background image for CFP

back_yfp = imcrop(YFP(:,:,j),back_rect1); Generate background image for YFP

mn1 = mean(mean(back_cfp)); Compute mean background for CFP

mn2 = mean(mean(back_yfp)); Compute mean background for YFP

cfp_filt = imfilter(CFP(:,:,j),H); %. 5X5 smoothing of the image for CFP

yfp_filt = imfilter(YFP(:,:,j),H); %. 5X5 smoothing of the image for CFP

cfp_back_sub = double(cfp_filt - mn1); Subtract background from CFP image

yfp_back_sub = double(yfp_filt - mn2); Subtract background from CFP image

leak = 0.4*cfp_back_sub; Computing leakage

PFRET = yfp_back_sub - leak; Get FRET intensity

tot_donor = PFRET + cfp_back_sub; Get Total donor intensity

FRET = PFRET./tot_donor; Estimate FRET Efficiency

FRET_Z(:,:,j) = FRET;

Tot_donor_Z(:,:,j) = tot_donor.*mask1; Get masked total donor intensity

end

Tot_don_lin = Tot_donor_Z(Tot_donor_Z>0);

mn = mean(Tot_don_lin); Get mean intensity of Total Donor

Generate background by rolling ball method

tot_don_back = imopen(Tot_donor_Z,strel('disk',25));

Tot_don_backsub = Tot_donor_Z - tot_don_back; Subtract Background

Tot_don_filt = medfilt3(Tot_don_backsub);

Tot_don_thresh = (Tot_don_filt>0.20*mn); Threshold the total donor

Thresholding FRET signal

FRET_thresh = zeros(row,col,N);

for k=1:N

FRET_thresh(:,:,k) = FRET_Z(:,:,k).*Tot_don_thresh(:,:,k);

end

Saving FRET values as a MATLAB matrix

filename = strcat('ATP_','Disc_',num2str(d,'%03d'),'.mat');

save(filename, 'FRET_thresh','Tot_donor_Z'); Save FRET and total donor matrix

count = count+1

FRET_lin = FRET_thresh(FRET_thresh>0);

mn_FRET = mean(FRET_lin);

All_disc_mean = cat(1,All_disc_mean,mn_FRET);

end
